# Clinical Utility of Eye Tracking in Assessing Distractibility in Children with Neurological Disorders or ADHD: A Cross-Sectional Study

**DOI:** 10.3390/brainsci12101369

**Published:** 2022-10-09

**Authors:** Dirk J. J. Sweere, Johan J. M. Pel, Marlou J. G. Kooiker, Johannes P. van Dijk, Elizabeth J. J. M. van Gemert, Petra P. M. Hurks, Sylvia Klinkenberg, R. Jeroen Vermeulen, Jos G. M. Hendriksen

**Affiliations:** 1 Kempenhaeghe Centre for Neurological Learning Disabilities, 5591 VE Heeze, The Netherlands; 2 Faculty of Health, Medicine and Life Sciences, Maastricht University, 6211 LK Maastricht, The Netherlands; 3 Erasmus Medical Centre, Vestibular and Oculomotor Research Group, Department of Neuroscience, 3000 CA Rotterdam, The Netherlands; 4 Academic Centre for Epilepsy Kempenhaeghe, 5591 VE Heeze, The Netherlands; 5 Berkenschutse School for Children with Special Needs, 5591 VE Heeze, The Netherlands; 6 Faculty of Psychology and Neuroscience, Maastricht University, 6211 LK Maastricht, The Netherlands; 7 Department of Neurology, Maastricht University Medical Centre, 6229 HX Maastricht, The Netherlands

**Keywords:** eye tracking, distractibility, assessment, ADHD, children, neurological disorders

## Abstract

This study aims to investigate distractibility quantified by recording and analyzing eye movements during task-irrelevant distraction in children with and without ADHD and in children with and without neurological disorders. Gaze behavior data and press latencies of 141 participants aged 6–17 that were collected during a computerized distraction paradigm with task-irrelevant stimuli (IDistrack) were analyzed. Children using attention-regulating medication were excluded from participation. Data were analyzed for subgroups that were formed based on the presence of neurological disorders and the presence of ADHD separately. Participants with ADHD and participants with neurological disorders spent less time fixating on the target stimuli compared to their peers without ADHD (*p* = 0.025) or their peers without neurological disorders (*p* < 0.001). Participants with and without ADHD had equal press latencies (*p* = 0.79). Participants with neurological disorders had a greater press latency compared to their typically developing peers (*p* < 0.001). Target fixation duration shows a significant association with parent-reported attention problems (*r* = −0.39, *p* < 0.001). We conclude that eye tracking during a distraction task reveals potentially valid clinical information that may contribute to the assessment of dysfunctional attentional processes. Further research on the validity and reliability of this paradigm is recommended.

## 1. Introduction

Children and adolescents with neurological disorders (e.g., neuromuscular disorders, cerebral palsy, traumatic brain injury) are often diagnosed with attention deficit hyperactivity disorder (ADHD) [1]. One of the symptoms, as described in the DSM-5 [2], is susceptibility to distraction. In a recent study, Forster and Lavie [3] proposed “an attention-distractibility trait” underlying this and other ADHD symptoms. These researchers presented data of healthy adults showing a positive association between the degree of task-irrelevant distractor interference (i.e., the observed slowing of visual reaction times caused by distracting stimuli that have no relation to the task at hand) and childhood ADHD symptom severity reported retrospectively. However, these results could not be replicated [4]. Nevertheless, these results show that distraction by task-irrelevant stimuli can be quantified as a specific attentional process. Assessment of task-irrelevant distraction during cognitive attention tasks contribute to the psychometric sensitivity of these tasks and may potentially be useful in the diagnosis and management of ADHD [5,6]. 

Traditionally, attentional processes during cognitive tasks are quantified in terms of the speed and accuracy with which task-relevant stimuli (i.e., stimuli that have to be processed for adequate completion of the task at hand) are processed and reacted upon. Similarly, in previous studies, assessment of distractibility is based on the speed and accuracy of information processing during task-irrelevant distraction [3,4,5,6]. Since the visual information that is actively processed can be derived from the direction of gaze [7,8,9], recording eye movements during visual distraction would provide a directly observable and therefore valid measure of the susceptibility to distraction. It can be argued that analysis of gaze behavior using eye tracking during cognitive tasks can provide deeper insight into the occurring attentional processes, including task-irrelevant distraction [10,11]. 

Two recent studies on this topic in adults showed enhanced correct ADHD classification rates for a validated task-irrelevant distractor test when gaze distribution information was added to the conventional outcome measures (i.e., speed and accuracy of information processing) [12,13]. These results were most prominent when visual distractors were used as compared to auditory distractors. This emphasizes the necessity of taking into account the effect of distractor modalities on the diagnostic effectiveness of such tests. At the level of visual distraction, it is important to take into account the possible mediating effects of visual distractor appearance. The visual properties and semantic properties (i.e., the meaningfulness in the context of daily life) of distractors used in distraction tests may influence the effectiveness of these tests (visual properties: [14,15], semantic properties: [11,16,17,18,19]).

Assessment of gaze behavior using eye tracking may contribute to a better understanding of distractibility in children with ADHD or neurological disorders. ADHD is presented as a heterogeneous clinical disorder, with patients presenting varying cognitive and behavioral symptoms [20]. Valid assessment of distractibility as a specific cognitive symptom of ADHD may therefore contribute to diagnosis, treatment, and management of symptoms in children with ADHD or neurological disorders in daily life. Analysis of gaze behavior through eye tracking during cognitive tasks has already proven to be beneficial in the understanding of attentional processes within a variety of disorders in children and adolescents (Autism [21]; Anxiety [22]; ADHD [23]). Furthermore, using eye movements instead of manual motor output in the assessment of attentional processes would make cognitive tasks suitable to a larger group of individuals with neurological disorders and motor disabilities (e.g., neuromuscular disorders). 

In the current study, we aimed to assess the clinical utility and validity of an eye-tracking-based cognitive task (IDistrack) we developed to assess the susceptibility of children to task-irrelevant distraction. In the first part of the study, we compared gaze behavior and manual press latencies during task-irrelevant distractor interference between children and adolescents with a formal ADHD diagnosis or neurological disorders and their non-ADHD or typically developing peers, respectively. We also assessed the association between gaze behavior data and parent-reported attention problems. In the second part of the study, we assessed the effects of distractor modality (i.e., auditory distractors, visual distractors with low semantic salience, visual distractors with high semantic salience, and combinations between the visual and auditory modalities) on the degree of task-irrelevant distraction. We hypothesize children and adolescents with ADHD or a neurological disorder to be most susceptible to task-irrelevant distraction and that the degree of task-irrelevant distraction is most prominent when visual distractors with high semantic salience are used.

## 2. Materials and Methods

### 2.1. Participants

A total of 177 Dutch children and adolescents aged between 6 and 17 years (mean = 9.67; SD = 2.76) were asked to participate in this study. Children with neurological disorders were recruited from two locations: Berkenschutse School for Special Education (SE, *n* = 37) and the Center for Neurological Learning Disabilities in Kempenhaeghe (NLD, *n* = 113). Typically developing children (TD, *n* = 27) were randomly recruited via coworkers at the Kempenhaeghe institute for epilepsy and sleep medicine. Depending on the age of the participants, either the parents/legal guardians (ages 6–11), the participant themselves (ages 16–17), or both the participant and the parents/legal guardians (ages 12–15) provided written informed consent. This is in line with the law on Medical Research Involving Human Subjects in The Netherlands. The study was approved by the local medical ethics committee in Kempenhaeghe (METC 18.01).

#### Inclusion and Exclusion Criteria

Children were included for participation in the SE and NLD group in the case that a neurological disorder was diagnosed by a neurologist. Children were included for participation in the TD group in the case that no neurological disorder was present, as indicated by the parents or legal guardians. Children were excluded from participation in this study in the following cases: (1) their age was below 6 or above 17 years; (2) medications prescribed for attention deficit disorders (e.g., methylphenidate) were being used.

### 2.2. Measurements and Procedures

On the day of testing, each participant completed the IDistrack task. A standard neuropsychological assessment was administered preceding the IDistrack task for participants in the NLD group as part of regular clinical care in the outpatient clinic. For the NLD group, additional test data included in this study consisted of the Attention Problems subscale of the Child Behavior Checklist (CBCL), which is a parent-reported questionnaire assessing the degree of attention problems in children and adolescents [24]. Parents of participating children in the TD group were asked to complete the CBCL questionnaire. For the SE group, CBCL data were not available. Information about use of psychopharmacologic drugs/medication and neurological and/or psychiatric diagnoses were collected for all participants. 

#### 2.2.1. Measurement of Parent-Reported Attention Problems

The Attention Problems subscale of the Child Behavior Checklist (CBCL) was used for quantification of parent-reported attention problems in the daily life functioning of the included participants. The CBCL is a parent-reported questionnaire assessing behavioral and emotional functioning in children aged 6–18 years (Cronbach’s Alpha for the total questionnaire is 0.97) [24]. The Attention Problems subscale used in the current study consists of 12 items that are answered according to a three-point scale reflecting the frequency of behavior mentioned in the item (i.e., Not at all, Sometimes, or Often). The outcome measure of the Attention Problems subscale is a T-score (M = 50, SD = 10) that is classified into three categories representing the degree of parent-reported attention problems (Normal: T ≤ 64, Borderline: 64 < T < 70, Clinical: T ≥ 70). In the current study, these score classifications have been used.

#### 2.2.2. ADHD Diagnosis

The diagnosis of ADHD in the NLD group was made on the basis of a standard protocol using the DSM-5, as described by Hendriksen et al. [1]. For the TD group, parents of the participants were asked for the presence of a formal ADHD diagnosis; for the SE group, information about ADHD diagnoses was requested from the school.

#### 2.2.3. The IDistrack Measurement Setup

The measurement setup consisted of a desktop computer, a 23-inch computer screen with a remote eye tracker attached to the center of the bottom edge of the screen, and a computer keyboard. The setup was put on a height-adjustable table in a low-stimulus room. Each participant sat in a comfortable chair with a viewing distance of the remote eye tracker (Tobii X2–60, Tobii, Danderyd Municipality, Sweden) between 45 cm and 75 cm, as indicated by the eye-tracking software. The height of the monitor was adjusted in such manner that the eyes of each participant were levelled with the center of the screen for optimal tracking of the eyes. The eye tracker recorded the participant’s eye movements at a sampling rate of 60 Hz, and compensated for free head movements. At the distance of 60 cm, the visual angle towards the monitor was approximately 30° × 24° and the system’s latency was in the order of 30 ms. The keyboard was positioned well within reach of each participant. After a standardized five-point calibration procedure, the IDistrack task was administered. The total test duration was four minutes. 

#### 2.2.4. The IDistrack Task 

**Figure 1:**
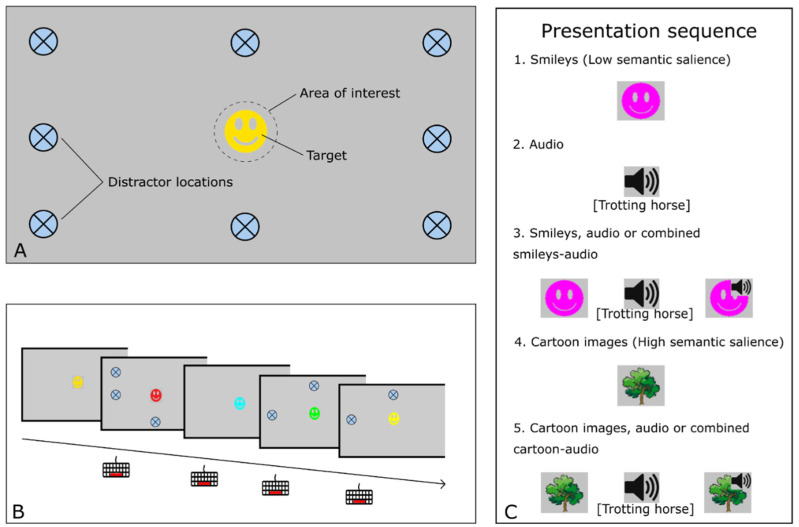
Schematic overview of the IDistrack task. (**A**) Task screen with the target stimulus presented in the center of the screen, which is surrounded by the target Area of Interest (AOI). Possible locations for distractor presentation are within the peripheral region of the screen. (**B**) Before each of the five presentation sequences, the participant is instructed to press the spacebar button as fast as possible after the target changes color. Meanwhile, visual distractors are presented and/or auditory distractors are played. The number of visual distractors that are simultaneously presented varies from one to five. (**C**) The IDistrack task consists of five presentation sequences that are presented in a fixed order: (1) Smileys (Low semantic salience); (2) Audio; (3) Smileys, Audio, or a combination of both; (4) Cartoon images (High semantic salience, e.g., a tree or a car); (5) Cartoon images, Audio, or a combination of both.

The IDistrack task is a reaction-time-based task-irrelevant distractor paradigm in which the participants were instructed to fixate on a smiley (target) that was presented in the center of the monitor (Figure 1A). The target was presented in the center of the screen for the entire duration of the task and changed color with semi-random timing. Meanwhile, visual distractors were shown near the edge of the monitor and/or auditory distractors were played at a standardized volume with semi-random timing and a duration varying between one and six seconds (Figure 1B). Distractors were either presented coinciding with or separately from the color change of the fixation target. The participant was instructed to confirm a detected color change by pressing the spacebar of the keyboard as quickly as possible. 

The test was divided into five presentation sequences that were separately and sequentially administered in a fixed order. Each presentation sequence contained one or multiple of the following five distractor modalities (see Figure 1C for an overview of the presented distractor modalities per presentation sequence): 1. Purple smiley shapes that are visually similar to the target (Smileys); 2. Easily recognizable sounds, e.g., a trotting horse or a honking car (Audio); 3. Purple smiley shapes combined with sounds (Smileys and Audio); 4. Cartoon images of everyday objects (Cartoons); 5. Cartoon images combined with sounds (Cartoons and Audio). In each sequence, color changes without any auditory or visual distractors were also added (No distractor modality). 

### 2.3. Data Analysis and Statistics

#### 2.3.1. Area of Interest and Post-Calibration of the Gaze Data

The center of the area of interest (AOI) (with a diameter of five degrees) and the center of the target stimulus (with a diameter of three degrees) were placed in the center of the screen. For every participant, raw gaze points as obtained via the eye tracker were plotted and visually inspected by the researcher (DS). A post-calibration procedure on the raw gaze points was performed when the central cluster of gaze points, assumed to be directed to the central AOI, was (partly) not within the AOI. This procedure was mainly performed when the calibration prior to the test was poorly conducted or when a child had altered their position with respect to the eye tracker too much. To this end, the center of this cluster was selected, as well as the intersection points of the horizontal and vertical gaze traces for each of the corners of the screen. The data points were then rescaled to the known location of the central target stimulus and the known peripheral distractor placeholders in order to account for gaze traces that were not attributable to distraction. 

#### 2.3.2. Feature Calculation

**Figure 2:**
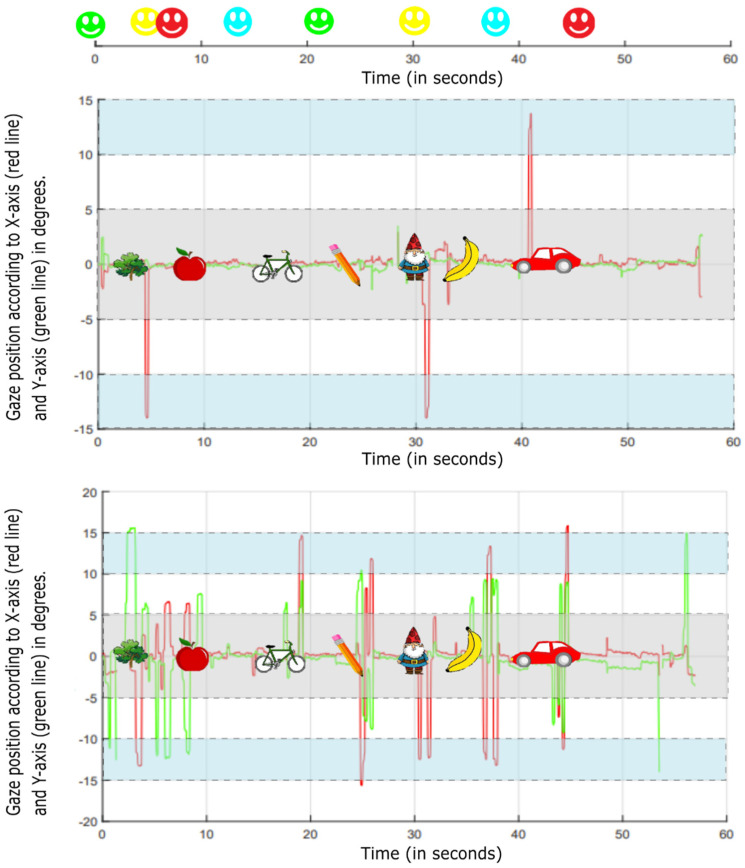
Two examples of a processed gaze signal during the fourth IDistrack presentation sequence of two participants (upper and bottom graph). Only Cartoon distractors were used in this presentation sequence. The *X*-axis represents the time in seconds. The *Y*-axis represents the *X*-co-ordinates (red line) and the *Y*-co-ordinates (green line). The grey area within the graph represents the location and surface of the area of interest (AOI) in the center of the screen. The timing of distractor presentation is visually represented within this area. The blue areas represent the peripheral location on the screen in which visual distractors were presented during the IDistrack task. The axis at the top of the figure shows the timing of the target color switches. This figure shows a difference in distractibility between the two participants as the gaze signal in the bottom graph shows more peaks into the distractor areas.

Recorded eye movement data were analyzed offline using a Matlab-based software program (Mathworks Inc., Natick, MA, USA). To analyze eye movements, gaze data were recalculated as a visual angle between gaze location and the center of the target area, using the average viewing distance of a child (see Figure 2 for an example of the processed gaze signal). The following events were marked: target color changes, distractors on, distractors off, and key presses. The eye movements were quantified in terms of the mean percentage of gaze time within the target area of interest (tAOI). The manual press latency (PL) was quantified as the time between the color switch and the first spacebar press with a minimum of 200 ms and a maximum of the time between the current and subsequent color switch. Manual press latencies that were the result of erroneously keeping the spacebar pressed down for a longer period starting before and ending after the color switch were excluded from the feature calculation process.

#### 2.3.3. Statistical Analysis

The data were analyzed using RStudio (RStudio Team, 2020). All parameters were averaged per participant and per distractor modality. Subgroup homogeneity for age was analyzed through a non-parametric Mann–Whitney test and subgroup homogeneity for gender; a diagnosis of ADHD and intake of psychoactive medication during IDistrack administration was analyzed using Chi-squared tests. A non-parametric equivalent to analysis of variance (nparLD [25]) was used to investigate the effects of task-irrelevant distraction on the mean percentage of gaze fixation time within the AOI (tAOI) and press latencies after a target color switch (PL), resulting in two separate models. Presence of a formal ADHD diagnosis was used as a between-subjects factor and distractor modality was used as a within-subjects factor (nparLD: F1-ld-F1 model). Post hoc analyses consisted of non-parametric pairwise comparisons (either Wilcoxon signed rank tests or Mann–Whitney tests) with Bonferroni corrections for multiple testing. Similar analyses were carried out for subgroups based on the presence of neurological disorders (TD, NLD, and SE subgroups). For the distractor modalities causing the most distraction, the associations between tAOI and the CBCL Attention Problems subscale were analyzed irrespective of subgroup using Spearman’s correlations. Statistical significance was set at *p* < 0.05.

## 3. Results

### 3.1. Inclusion Criteria for Statistical Analysis

Eye tracking data of participants were included for analyses based on data quality and comprehension of instruction. These parameters were quantified as the percentage of total gaze samples collected and the number of spacebar presses. To include reliable gaze responses, a cut-off point for the percentage of gaze samples was set at 50% based on visual inspection. The cut-off point for average number of spacebar presses per presentation sequence was established on 20 presses based on the maximum presence of 16 events per sequence (8 target color changes and 8 distractor events). This means that in case the participant pressed the spacebar after all target events and all distractor events, data are still included for analysis. These criteria resulted in the exclusion of 36 of the total of 177 participants: 16 of the 37 SE children (43%), 18 of the 113 NLD children (16%), and 2 of the 27 TD children (7%). The mean age (M = 7.50, SD = 2.24) and mean IQ (M = 78.32, SD = 19.10) of the excluded participants were significantly lower compared to the participants that were included for further analysis (Age: W = 1112, *p* < 0.001; IQ: W = 916, *p* = 0.01)

### 3.2. Homogeneity of Subgroups

A non-parametric Mann–Whitney test was conducted to check for differences in age between the ADHD and non-ADHD subgroups. No significant difference was found (W = 1928, *p* = 0.30). Chi-squared tests for homogeneity show an unequal distribution of gender over the ADHD subgroups (Χ^2^ (1, *n* = 141) = 3.99, *p* = 0.046) and an equal distribution of psychoactive medication (X^2^(1, *n* = 141) < 0.001, *p* = 1). Available IQ scores for participants with ADHD (M = 87.64, SD = 13.21, *n* = 36) and without an ADHD diagnosis (M = 88.55, SD = 16.17, *n* = 58) are similar (Mann–Whitney test: W = 1136, *p* = 0.48). See Table 1 for an overview of the demographics after inclusion for analysis.

A non-parametric Kruskal–Wallis test was conducted to check for differences in age between the SE, NLD, and TD subgroups. Besides the mean age of the NLD participants being significantly higher than the TD participants (H(2) = 6.59, *p* = 0.04), these subgroups did not differ in age. Chi-squared tests for homogeneity show an equal distribution of gender over the SE, NLD, and TD subgroups (Χ^2^ (2, *n*=141) = 0.01, *p* = 0.995) but no equal distribution of ADHD diagnoses (X^2^ (2, *n* = 141) = 11.93, *p* = 0.003) and no equal distribution of psychoactive medication intake (X^2^ (2, *n* = 141) = 20.66, *p* < 0.001). IQ testing of the NLD and SE neurological disorder subgroups revealed a low average IQ for both groups (NLD: M = 89.42, SD = 15.14, *n* = 74; SE: M = 83.70, SD = 14.20, *n* = 20), and no significant difference was found (Mann–Whitney test: W = 574, *p* = 0.13). The TD group is assumed to have an average IQ, as they attended regular schooling and no IQ data were available for this group.

### 3.3. IDistrack Outcome Measures in Relation to a Clinical ADHD Diagnosis

#### 3.3.1. Time in Area of Interest (tAOI)

**Figure 3:**
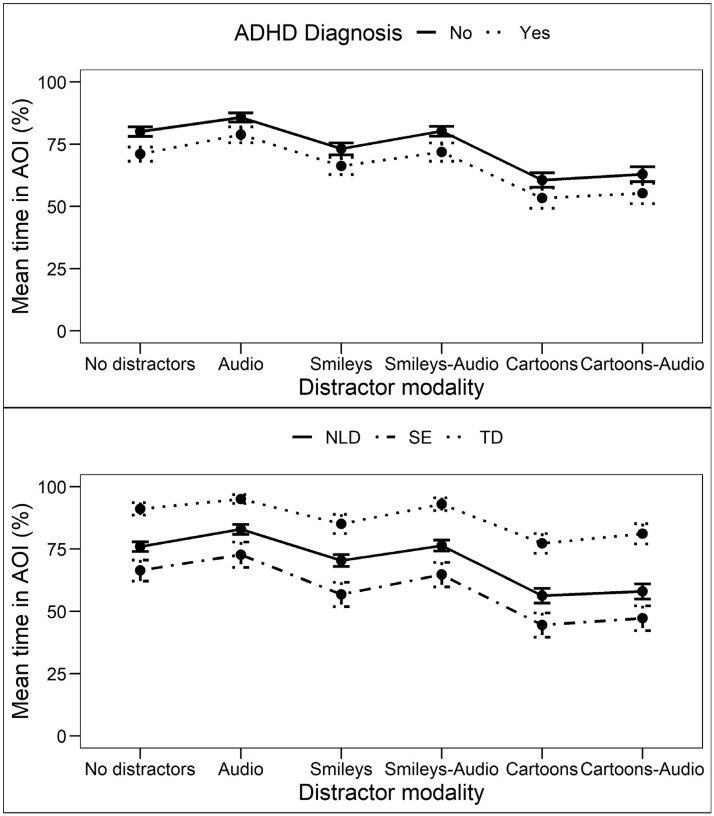
Mean time in AOI (tAOI) in percentage for subgroups based on presence of a formal ADHD diagnosis (upper) and neurological disorder (bottom). NLD—Neurological Learning Deficit; SE—Special Education; TD—Typically Developing. Error bars indicate the standard error of the mean (M ± SEM).

A significant main effect of ADHD diagnosis was found (F_ATS_ = 5.01, *p* = 0.025), indicating that children diagnosed with ADHD spent less time looking in the AOI, compared to their non-ADHD peers (Figure 3). Additionally, a significant main effect of distractor modality on tAOI was found (F_ATS_ = 92.33, *p* < 0.001). Post hoc non-parametric pairwise comparisons show that the Cartoon and the combined Cartoon–Audio modalities correspond to significantly lower scores than all other modalities (*p* < 0.001 for all comparisons). No significant interaction effect of ADHD diagnosis by distractor modality was found (F_ATS_ = 0.58, *p* = 0.66).

#### 3.3.2. Press Latencies (PL)

**Figure 4:**
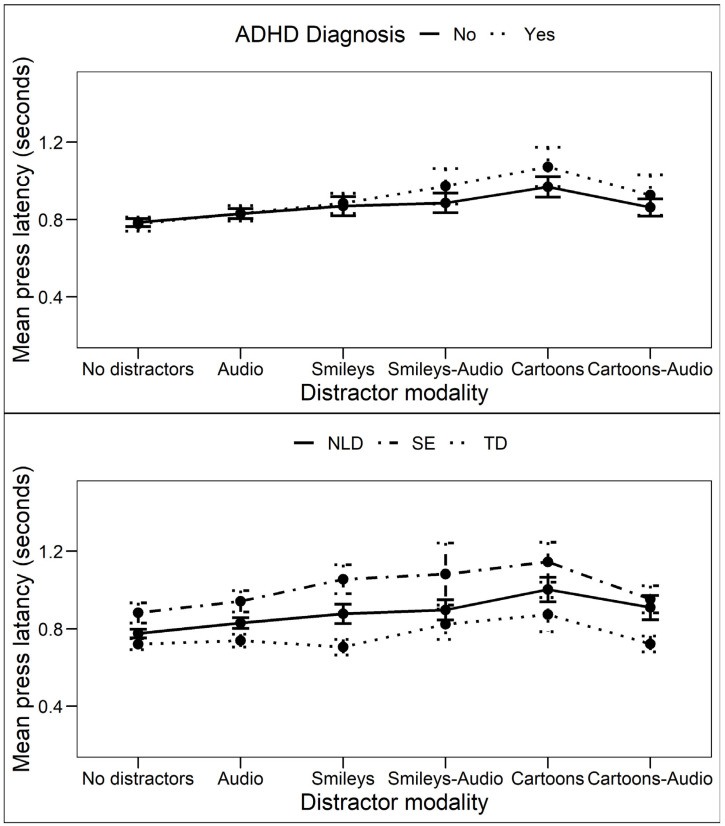
Mean manual press latency (PL) in seconds for subgroups based on presence of a formal ADHD diagnosis (upper) and neurological disorder (bottom). NLD—Neurological Learning Deficit; SE—Special Education; TD—Typically Developing. Error bars indicate the standard error of the mean (M ± SEM).

No significant main effect of ADHD diagnosis on PL was found (F_ATS_ = 0.07, *p* = 0.79), indicating that children with and without ADHD had equal manual press latencies (Figure 4). A significant main effect of the Distractor modality on PL was found (F_ATS_ = 5.69, *p* < 0.001). Post hoc non-parametric comparisons show significantly higher manual press latencies for the Cartoon modality compared to all other modalities without Bonferroni correction (*p* < 0.05 for all comparisons). After Bonferroni correction for multiple testing, only the difference with the combined Smiley–Audio modality became non-significant. No significant interaction effect of ADHD diagnosis by distractor modality was found (F_ATS_ = 0.74, *p* = 0.57).

### 3.4. IDistrack Outcome Measures in Relation to Neurological Disorder

#### 3.4.1. Time in Area of Interest (tAOI)

A significant main effect of subgroup on tAOI was found (F_ATS_ = 24.01, *p* < 0.001). Post hoc non-parametric pairwise comparisons show that the TD group spent significantly more time in the AOI compared to the NLD group and the SE group. Furthermore, the NLD group spent significantly more time in the AOI than the SE group (*p* < 0.001 for all comparisons; see Figure 3). Additionally, a significant main effect of distractor modality on tAOI was found (F_ATS_ = 86.69, *p* < 0.001). Non-parametric post hoc pairwise comparisons show that, irrespective of subgroup, the participants spent significantly less time looking in the AOI when Cartoon distractors or combined Cartoon–Audio distractors were presented, compared to all other distractor modalities (*p* < 0.001 for all comparisons). This indicates that irrespective of participant subgroup, visual distractors with high semantic salience have the strongest distracting effect. The results show no significant interactions of participant subgroup by distractor modality (F_ATS_ = 0.66, *p* = 0.67). 

#### 3.4.2. Press Latencies (PL)

A significant main effect of subgroup on PL was found (F_ATS_ = 7.63, *p* < 0.001). Post hoc non-parametric pairwise comparisons show that the TD group had significantly lower manual press latencies compared to the NLD group and the SE group. Furthermore, the NLD group had significantly lower manual press latencies than the SE group (*p* < 0.01 for all comparisons; see Figure 4). Additionally, a significant main effect of distractor modality on PL was found (F_ATS_ = 4.80, *p* < 0.001). Post hoc non-parametric pairwise comparisons show that manual press latencies for the Cartoon modality were significantly higher than for the other modalities, but after Bonferroni correction, the difference with the combined Smileys–Audio modality became non-significant. The results show no significant interactions of participant subgroup by distractor modality (F_ATS_ = 1.37, *p* = 0.21). 

### 3.5. Correlation of Eye Movement Responses with Parent-Reported Attention Problems

The associations between tAOI of the most effective distractor modalities causing the most task-irrelevant distraction (Cartoons and combined Cartoon–Audio) and a validated behavioral questionnaire assessing parent-reported attention problems (CBCL) were analyzed. Data of the CBCL parent-reported questionnaire were available for participants in the NLD and TD subgroups (*n =* 88). The results show significant negative Spearman’s correlations between the classification score on the CBCL Attention Problems subscale (i.e., a T-score corresponding to a normal, borderline, or clinical classification of attention problems) and tAOI in the Cartoon modality (r_s_ = −0.36, *p* < 0.001, *n* = 88) and the combined Cartoon–Audio modality (r_s_ = −0.39, *p* < 0.001, *n* = 88). 

## 4. Discussion

To the best of our knowledge, this is the first study reporting on a computer-assisted assessment paradigm for children and adolescents of six years and older assessing task-irrelevant distraction on the basis of eye tracking data. We developed an easy-to-administer, simple, and efficient computerized testing procedure, wherein manual press latency and eye movements are recorded during visual and auditory distraction in a simple visual reaction time task. The testing procedure requires minimal verbal instruction and requires minimal manual motor output from the participants. This makes the test procedure suitable for a wide range of children and adolescents with both minor and major motor disabilities. 

When comparing the gaze behavior of the subgroups during the IDistrack task, the results are as expected: children and adolescents with ADHD show more distractibility compared to children and adolescents without ADHD. Furthermore, similar differentiation is found between the subgroups based on the presence of neurological disorders: children and adolescents with neurological disorders show more distractibility compared to typically developing individuals. These findings are in line with the elevated comorbidity rates of attention deficit disorders in children and adolescents with neurological disabilities [1]. 

Our data show a spectrum of distractibility problems that are similar to those described by Forster and Lavie [3]. This implies that distractibility may be clinically presented in children with ADHD and in children with neurological disorders as a dysfunctional attentional process. However, in our study this is only consistently reflected in the gaze behavior, as we did not observe the expected differences in manual press latencies between the ADHD and non-ADHD group. This underlines the clinical utility and added value of recording eye movements in this clinical population.

Regarding the different distractor modalities, the results are partly as expected. Cartoon distractors (which have a high semantic load and are semantically meaningful in the context of daily life: e.g., a tree or a bike) caused the most distraction compared to auditory distractors and visual distractors with low semantic load (e.g., a single-color smiley shape). This is in line with previous attempts with eye-tracking-based distractor paradigms in an adult population [12,13] and literature on task-irrelevant distraction [16,17,18,19]. In our study, participants with ADHD or neurological disabilities were not disproportionately affected by changes in distractor modality as no interaction effects were found. Nevertheless, IDistrack outcome measures for the semantically meaningful Cartoon modality show a significant, moderate association with parent-reported attention problems (CBCL). Semantically meaningful distractors are therefore sensitive in the assessment of distractibility through eye tracking.

Based on the results of our study, we conclude that eye tracking during a task-irrelevant distractor paradigm is a feasible, efficient, and valid method for the measurement of distractibility in children. Evidence of elevated levels of distractibility in children with attention deficit disorders or neurological disorders is provided. These findings contribute to the notion of an attention-distractibility trait that is clinically relevant in the assessment of cognitive attention problems in children, and that assessment of distractibility should be taken into account in neuropsychological evaluations of children and adolescent with neurological disorders. The IDistrack paradigm has been shown in the current study to be a suitable and valid paradigm for clinical assessment of distractibility. Eye distraction data may enhance the diagnostic precision of a neuropsychological assessment for the assessment of ADHD in children and adolescents. Further research is needed.

### 4.1. Limitations

The current study may have several shortcomings. First, the IDistrack paradigm lacks an initial set of trials without the presence of distractors that serves as a baseline measurement for manual press latency. Comparing manual press latencies during IDistrack with this baseline would enhance the validity by eliminating confounding factors, such as cognitive processing speed. Second, because of the selection of patients with epilepsy, a considerable proportion (29%) of the SE group used anti-epileptic drugs (AEDs) during the administration of the IDistrack task. Cognitive side-effects of the AEDs could have been a confounding factor by causing high press latencies in this group [26]. Moreover, possible epileptic events (e.g., absences) during task administration were not recorded. For the ADHD and non-ADHD groups, psychoactive drug intake was equally distributed and no significant differences in manual press latency were found. Third, the cut-off point for minimal eye tracking data quality was set at a maximum of 50% gaze data loss during task administration. Though these amounts of data loss are acceptable for the current research purposes [27], this cut-off point remains arbitrary. 

### 4.2. Future Research

Further research is needed on the clinical utility of this procedure. In order to obtain normative scores for the purpose of discriminating between normal and disturbed distractibility levels, data on a larger group of children need to be collected and the effects of age on distractibility need to be investigated [28]. Furthermore, the clinical relevance of the paradigm should be investigated by comparing eye tracking data in children with ADHD during an off and on period of attention-regulating medication. This may be a simple and quick method for medication monitoring in ADHD and needs further research. Our research group is currently collecting these data. Further research on the reliability, criterion validity, and construct validity in a greater group of typically developing children and children with neurological disorders should be conducted in order to establish the psychometric quality of the IDistrack task. For this purpose, we suggest enhancing the current IDistrack task by adding a baseline measurement without distraction before starting the distraction procedure, using only visual distractors that are meaningful in the context of daily life.

## 5. Conclusions

To the best of our knowledge, the current study is the first to use eye tracking data in the formal assessment of distractibility in children with ADHD and children with neurological disorders. Eye-tracking technology facilitates accurate and valid assessment of distractibility as a specific attentional process in children. A proof of concept for this eye tracking paradigm has been provided in a clinical population. Data are promising regarding the validity and feasibility of this eye tracking paradigm in clinical practice and merit further research. As the paradigm requires minimal motor function, minimal verbal instruction, and minimal time for the task to be performed, it is a promising instrument to be used in a heterogeneous group of children with and without neurological disorders. 

## Figures and Tables

**Table 1:** Demographic characteristics of the sampled subgroups (based on a present ADHD diagnosis or neurological disorder).

	ADHD Diagnosis		Subgroup		
	Yes	No	SE	NLD	TD
N	45	96	21	95	25
Gender (%):					
Male	80%	62%	67%	67%	68%
Female	20%	38%	33%	33%	32%
Age range	6–16	6–17	7–12	6–17	6–15
Mean age (SD)	10.56 (2.54)	10.06 (2.64)	9.81 (1.54)	10.59 (2.70)	9.16 (2.70)
ADHD diagnosis (%):	100%	0%	29%	40%	4%
Psychoactive medication (%):					
Anti-epileptic drugs	7%	5%	29%	2%	0%
SSRI’s	0%	1%	0%	1%	0%

ADHD—Attention Deficit/Hyperactivity Disorder; SE—Special Education; NLD—Neurological Learning Deficit; TD—Typically developing; SSRI—Selective Serotonin Reuptake Inhibitor.

## Data Availability

The data presented in this study are available upon reasonable request from the corresponding author. The data are not publicly available due to privacy restrictions.
